# Identification of PANoptosis-related genes in lung cancer and investigation of their role mechanisms in the immune microenvironment

**DOI:** 10.1007/s12672-026-04739-1

**Published:** 2026-02-28

**Authors:** Shuang Han

**Affiliations:** Department of Neonatology, The First Hospital of China Medical University, Shenyang, Liaoning China

**Keywords:** Lung cancer, PANoptosis-related genes, Immune infiltration, Biomarkers, Therapeutic targets, Immune microenvironment

## Abstract

**Introduction:**

Lung cancer remains a leading cause of cancer-related death globally, with high malignancy and poor prognosis. PANoptosis, a novel programmed cell death pathway combining apoptosis, pyroptosis, and necroptosis, has been increasingly implicated in disease pathogenesis, yet its role in lung cancer is poorly understood. This study integrated bulk and single-cell RNA-seq data from TCGA and GEO databases to identify and validate PANoptosis-related genes in lung cancer. We identified eight key genes (DAPK2, CAV1, PDK4, IL3RA, NOTCH1, CHMP4B, IRAK1, and SFN) that are significantly dysregulated in tumors and correlated with prognosis. Functional enrichment analysis implicated these genes in cell adhesion, cytokine signaling, necrotic pathways, and cell cycle regulation. Furthermore, immune infiltration analysis suggested roles for CHMP4B and IRAK1 in modulating the tumor immune microenvironment. Experimental validation via qPCR and IHC confirmed differential expression of these genes in cell lines and clinical samples. Drug sensitivity analysis also linked CHMP4B and PDK4 to response to targeted agents like Gefitinib. In conclusion, our results reveal the significance of PANoptosis-related genes in lung cancer pathogenesis and highlight their potential as prognostic biomarkers and therapeutic targets.

**Background:**

Lung cancer is among the leading causes of cancer-related morbidity and mortality worldwide, characterized by high aggressiveness, early metastasis, and poor prognosis. PANoptosis, a recently recognized form of programmed cell death that integrates apoptosis, pyroptosis, and necroptosis, has been implicated in various diseases. However, comprehensive studies investigating PANoptosis-related genes in lung cancer remain limited, and their functional roles in tumorigenesis and progression are not fully understood.

**Methods:**

In this study, we integrated transcriptomic data from The Cancer Genome Atlas (TCGA) and Gene Expression Omnibus (GEO) databases with single-cell RNA sequencing analysis to identify PANoptosis-related genes involved in lung cancer. A combination of differential gene expression analysis, functional enrichment analysis, immune infiltration profiling, and drug sensitivity prediction was performed to explore the biological significance and therapeutic potential of these genes. Furthermore, we validated the expression patterns of key genes using quantitative real-time PCR (qPCR) and immunohistochemistry (IHC) in lung cancer cell lines and tissue specimens.

**Results:**

Eight PANoptosis-related genes (DAPK2, CAV1, PDK4, IL3RA, NOTCH1, CHMP4B, IRAK1, and SFN) were identified as being significantly associated with lung cancer. Their expression levels were notably altered in tumor tissues compared to normal controls and were significantly correlated with patient prognosis. Functional enrichment analysis revealed these genes were mainly involved in cell adhesion, cytokine signaling, necrosis-related pathways, and cell cycle regulation. Notably, CHMP4B and IRAK1 showed distinct expression patterns in different immune cell populations, indicating their potential roles in shaping the tumor immune microenvironment. Importantly, qPCR and IHC analyses confirmed the differential expression of these genes across lung cancer cell lines and clinical specimens. Drug sensitivity analysis further suggested that genes such as CHMP4B and PDK4 were associated with the response to targeted therapies including Gefitinib.

**Conclusion:**

This study provides a comprehensive landscape of PANoptosis-related gene dysregulation in lung cancer and highlights their potential as prognostic biomarkers and therapeutic targets. The integrated bioinformatics analysis combined with experimental validation supports their functional relevance in lung cancer development and treatment.

**Supplementary Information:**

The online version contains supplementary material available at 10.1007/s12672-026-04739-1.

## Introduction

Lung cancer stands as one of the malignant tumors with the highest incidence and mortality rates globally, characterized by its high aggressiveness and metastatic propensity, which results in a poor prognosis for patients [1]. According to statistics from the World Health Organization, the 5-year survival rate for lung cancer is less than 20%. This grim statistic is primarily attributed to the fact that most patients are diagnosed at an advanced stage, missing the optimal window for treatment. In recent years, with the continuous deepening of research into the molecular mechanisms of lung cancer, the role of apoptosis-related genes in the oncogenesis and progression of lung cancer has gradually garnered attention. Apoptosis, a programmed cell death process, is crucial for maintaining tissue homeostasis and inhibiting tumorigenesis [2]. However, traditional apoptosis research has primarily focused on single pathways or a limited number of genes, neglecting the complexity and diversity of cell death processes.

Recently, PANoptosis, a novel form of cell death, has emerged, integrating various modes of cell death such as apoptosis, necrosis, and pyroptosis, and is believed to play pivotal roles in numerous diseases [3]. The discovery of PANoptosis provides a new perspective for understanding the complexity of cell death. Unlike traditional apoptosis, PANoptosis involves the crosstalk and synergistic effects of multiple signaling pathways, including caspase-dependent apoptotic pathways, RIPK1/RIPK3-dependent necrotic pathways, and GSDMD-dependent pyroptotic pathways [4–6]. The synergistic activation of these pathways can amplify and accelerate cell death, thereby playing a crucial role in pathophysiological processes. In oncology, PANoptosis-related genes may affect tumor cell survival, proliferation, and immune evasion by regulating cell death signaling pathways. However, systematic studies on PANoptosis-related genes in lung cancer are still relatively scarce.

Significant progress has been made in the study of the molecular mechanisms of lung cancer, particularly in the research on driver gene mutations, such as mutations in EGFR, ALK, and ROS1, which have provided important bases for targeted therapy. Nevertheless, these driver gene mutations can only explain part of the oncogenesis mechanisms of lung cancer, and a large number of lung cancer patients still lack effective targeted therapeutic options. Additionally, the emergence of immunotherapy has brought new hope for the treatment of lung cancer [7], but the efficacy of immunotherapy varies significantly among different patients, suggesting that the immune microenvironment of lung cancer plays a crucial role in treatment response [8, 9]. Therefore, in-depth research into the molecular mechanisms of lung cancer, especially the regulatory networks of cell death-related genes, is of great significance for the development of new therapeutic strategies.

In recent years, with the development of high-throughput sequencing technology, single-cell sequencing technology has provided new tools for studying the molecular mechanisms of lung cancer. Single-cell sequencing technology can dissect the cellular heterogeneity within lung cancer tissues, revealing the roles of different cell types in the oncogenesis and development of lung cancer [10]. Through single-cell sequencing analysis, immune cell subtypes, tumor cell subclones, and stromal cell types within lung cancer tissues can be identified, thereby providing insights into the cellular composition and molecular characteristics of lung cancer [11, 12]. Additionally, single-cell sequencing technology can be used to study the expression patterns of PANoptosis genes in different cell types, revealing their cell-specific mechanisms of action in lung cancer. For example, through single-cell sequencing analysis, it can be found that certain PANoptosis genes are highly expressed in specific immune cell subtypes, which may be closely related to the immune microenvironment of lung cancer.

In terms of lung cancer treatment, traditional chemotherapy and radiotherapy can control tumor progression to a certain extent, but they have significant side effects and limited efficacy in patients with advanced lung cancer. In recent years, the emergence of targeted therapy and immunotherapy has brought new hope for the treatment of lung cancer. Targeted therapy can significantly prolong the survival of patients by specifically inhibiting driver gene mutations in tumor cells. However, the issue of drug resistance to targeted therapy is a major challenge at present. Immunotherapy attacks tumor cells by activating the patient’s immune system, providing new treatment options for patients with advanced lung cancer [13]. Nevertheless, the efficacy of immunotherapy varies significantly among different patients, suggesting the need for further research into the immune microenvironment of lung cancer to improve the efficacy of immunotherapy [14, 15]. Therefore, research on PANoptosis-related genes not only contributes to a deeper understanding of the molecular mechanisms of lung cancer but also may provide potential targets for the development of new therapeutic strategies.

The aim of this study is to identify PANoptosis-related genes in lung cancer through systematic bioinformatics analysis, reveal their mechanisms of action in the oncogenesis and progression of lung cancer, and evaluate their application potential in the diagnosis, prognosis, and treatment of lung cancer.

## Materials and methods

### Transcriptome data acquisition and processing

Eight lung cancer-related datasets were obtained from the Gene Expression Omnibus (GEO) database, namely GSE32665 (*n* = 179), GSE32863 (*n* = 116), GSE33532 (*n* = 100), GSE43458 (*n* = 110), GSE63459 (*n* = 65), GSE74706 (*n* = 36), GSE75037 (*n* = 166), and GSE13481 (*n* = 74), totaling 846 samples. Each dataset was standardized using log2 transformation. The Combat (3.54.0) method was employed to remove batch differences. Additionally, information on whether each sample was normal or cancerous was obtained for subsequent use. Furthermore, expression data and clinical survival data for lung adenocarcinoma (LUAD) and lung squamous cell carcinoma (LUSC) were downloaded from The Cancer Genome Atlas (TCGA) for subsequent prognostic analysis. Among them, GSE75037, GSE63459, GSE43458, GSE32665, GSE74706, GSE134381, GSE32863 represents the Training cohorts, and GSE33532 represents the Validation Cohorts. All these data can be download from GEO database (https://www.ncbi.nlm.nih.gov/geo/) and TCGA (https://cancergenome.nih.gov/).

### Single-cell data acquisition and analysis

The GSE131907 dataset was obtained from the GEO database, with non-tumor samples excluded. The data were analyzed using scanpy (Version 1.11.4) and R software (Version 4.4.3). Quality control criteria included: each cell expressing at least 200 genes; each gene expressed in at least 3 cells; retention of cells with a mitochondrial gene proportion of less than 10%; and retention of cells with a total count of less than 40,000. Subsequently, 3,000 highly variable genes were selected for principal component analysis (PCA). Sample integration was performed using bbknn (Version 1.6.0). Initially, major immune-related cell types were identified: B cells, T cells, and myeloid cells. For each of these three cell types, highly variable genes were recalculated, PCA was performed, and clustering was conducted to identify cell subtypes. Cell types expressing the target genes were identified, and differential gene expression analysis was performed between high- and low-expression groups which the expression level is higher or lower than the median, followed by enrichment analysis and single-cell prognostic analysis.

### Acquisition of PANoptosis-related genes

A novel model of PANoptosis-related genes was utilized for enhanced prognosis and immune status prediction in kidney renal clear cell carcinoma, serving as a reference for acquiring PANoptosis-related genes in our study.

### Drug-related data acquisition and analysis

Drug-gene interaction data were downloaded from the DSigDB database (https://dsigdb.tanlab.org/DSigDBv1.0/) for drug-gene enrichment analysis. For significantly enriched drugs, drug structures were downloaded from the PubChem database (https://pubchem.ncbi.nlm.nih.gov/), and protein structures were downloaded from the RCSB database (https://www.rcsb.org/). Molecular docking was performed between drugs and proteins.

### Identification of PANoptosis-related DEGs in lung cancer

For the GEO datasets, samples were divided into a control group (*n* = 370) and an experimental group (*n* = 476) based on their cancer status. Differentially expressed genes (DEGs) between the two groups were identified using the limma package. The obtained DEGs were intersected with PANoptosis-related genes, resulting in 21 differential genes related to PANoptosis. Subsequently, significant genes were screened through univariate logistic regression, yielding 21 genes. These 21 genes were then used as features to construct random forest and LASSO classification models for feature selection. The genes selected by both methods were intersected, ultimately resulting in 8 genes as the final lung cancer-related PANoptosis genes, named as PANoptosis-related DEGs in lung cancer.

### Enrichment analysis

The Kyoto Encyclopedia of Genes and Genomes (KEGG) and the Gene Ontology (GO) enrichment analyses were performed using clusterProfiler, and the gene set enrichment analysis (GSEA) was also conducted using clusterProfiler. Only results with an adjusted p-value < = 0.05 were considered significant.

### Prognostic analysis

To evaluate the prognostic value of the identified PANoptosis-related DEGs, a multivariate Cox proportional hazards regression model was constructed. The expression levels of the eight key genes (DAPK2, CAV1, PDK4, IL3RA, NOTCH1, CHMP4B, IRAK1, and SFN) were used as covariates. Based on the linear predictor derived from the Cox model, a risk score was calculated for each patient. Patients were then stratified into high-risk and low-risk groups using the median risk score as the cutoff point. Kaplan-Meier survival analysis was subsequently performed to compare the overall survival (OS) differences between the two groups, and the statistical significance was assessed using the log-rank test. This analysis was conducted separately for LUAD and LUSC cohorts from the TCGA database to investigate potential subtype-specific prognostic implications.

### Immunohistochemical images

To validate the protein expression patterns of the identified genes, immunohistochemical staining images were systematically retrieved from the Human Protein Atlas (HPA) database (https://www.proteinatlas.org/). The staining results for each target gene in both lung cancer tissues and normal lung tissues were reviewed and compared. This qualitative assessment at the protein level provided independent corroboration of the transcriptomic findings, offering visual evidence of differential protein expression (e.g., staining intensity and cellular localization) between malignant and normal states.

### Immune infiltration analysis

The single sample GSEA (ssGSEA) was used to score immune infiltration in samples. For feature genes, the Spearman correlation coefficient between gene expression and immune infiltration scores was calculated to assess their correlation.

### Quantitative real-time PCR (qRT-PCR)

Total RNA was extracted from BEAS-2B, PC9, A549, SK-MES-1, and NCI-H226 cell lines using the Takara MiniBEST Universal RNA Extraction Kit (Takara Bio, Japan). cDNA was synthesized from 1 µg RNA using the PrimeScript™ RT reagent Kit with gDNA Eraser (Takara Bio). qRT-PCR was performed with TB Green^®^ Premix Ex Taq™ II (Takara Bio). GAPDH was used as the internal control. Relative gene expression was calculated using the 2^(-ΔΔCt) method. All experiments were performed in triplicate (primer sequences are shown in Supplementary Table 1).

### Statistical analysis

The Wilcoxon rank-sum test was used to determine whether there were differences between two groups. The Spearman correlation coefficient was used to calculate correlations between two groups. Unless otherwise specified, an adjusted p-value < = 0.05 was considered significant. The log-rank test was used to calculate p-values for survival curves.

## Results

### Differential gene identification

**Fig. 1:**
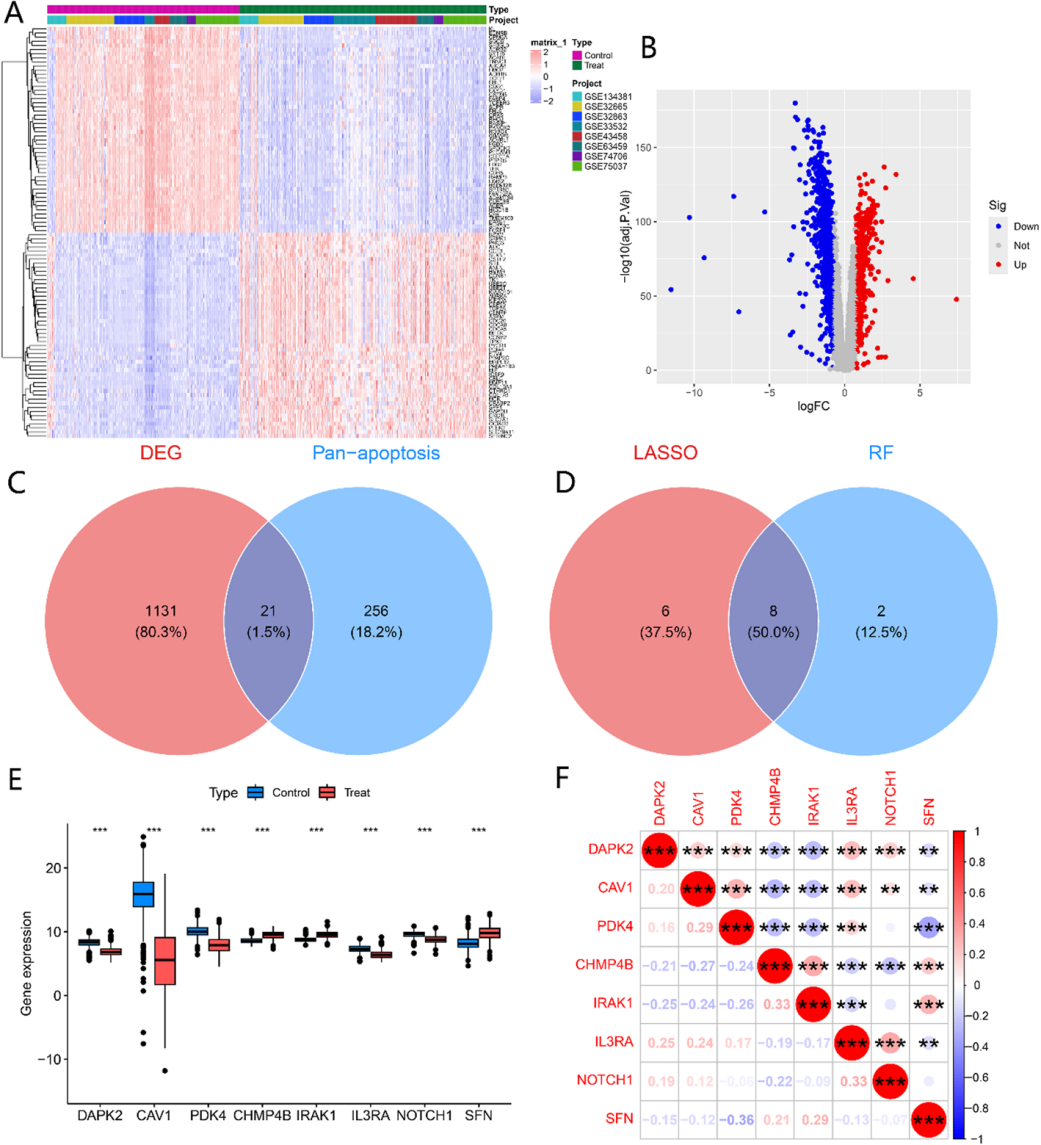
Comprehensive Analysis and Identification of PANoptosis-related Genes in Lung Cancer. **A** Heatmap Illustrating the Expression Patterns of the Top 50 Upregulated and Downregulated Genes. **B** Volcano Plot Differentiating Cancerous and Normal Groups. **C** Overlap between Differentially Expressed Genes and PANoptosis-Related Genes. **D** Comparative Evaluation of Feature Genes Identified by LASSO and Random Forest Models. **E** Expression Profiles of the Final Selected Genes in Cancerous and Normal Groups. **F** Correlation Analysis of the Expression of the Final Selected Genes in the Cancerous Context

The collected datasets were grouped into cancer and normal samples, and differential genes between cancer and normal samples were identified using the criteria of abs(logFC) > = 2 and adj.p < = 0.05. Figure 1A displays the top 50 upregulated and downregulated genes, respectively, revealing significant differences in gene expression between lung cancer tissues and normal tissue samples. Figure 1B presents the corresponding volcano plot. The PCA of the two components in each dataset is shown in Supplementary Fig. 1A, B. A total of 1,152 differential genes were identified. An intersection analysis was performed between these differential genes and the collected 277 PANoptosis-related genes, resulting in 21 PANoptosis-related differential genes (Fig. 1C). Subsequently, univariate logistic regression was employed to further screen the genes, and no genes were excluded. Lasso regression and random forest models were then constructed, with the optimal lambda for Lasso cross-validation shown in Supplementary Fig. 2A-C and the importance of gene selection in the random forest model shown in Supplementary Fig. 2D. Feature selection was conducted on these 21 genes, and the intersection of the results from both methods yielded 8 genes (Fig. 1D). Figure 1E exhibits the gene expression patterns of these 8 genes in normal and cancer groups. As shown in Fig. 1E, DAPK2, CAV1, PDK4, IL3RA, and NOTCH1 genes were significantly downregulated in lung cancer tissues, whereas CHMP4B, IRAK1, and SFN genes were significantly upregulated. Figure 1F displays the expression correlations of these 8 genes in cancer. The chromosomal locations of these 8 feature genes are presented in Supplementary Fig. 2E.

### Immunohistochemical staining

**Fig. 2:**
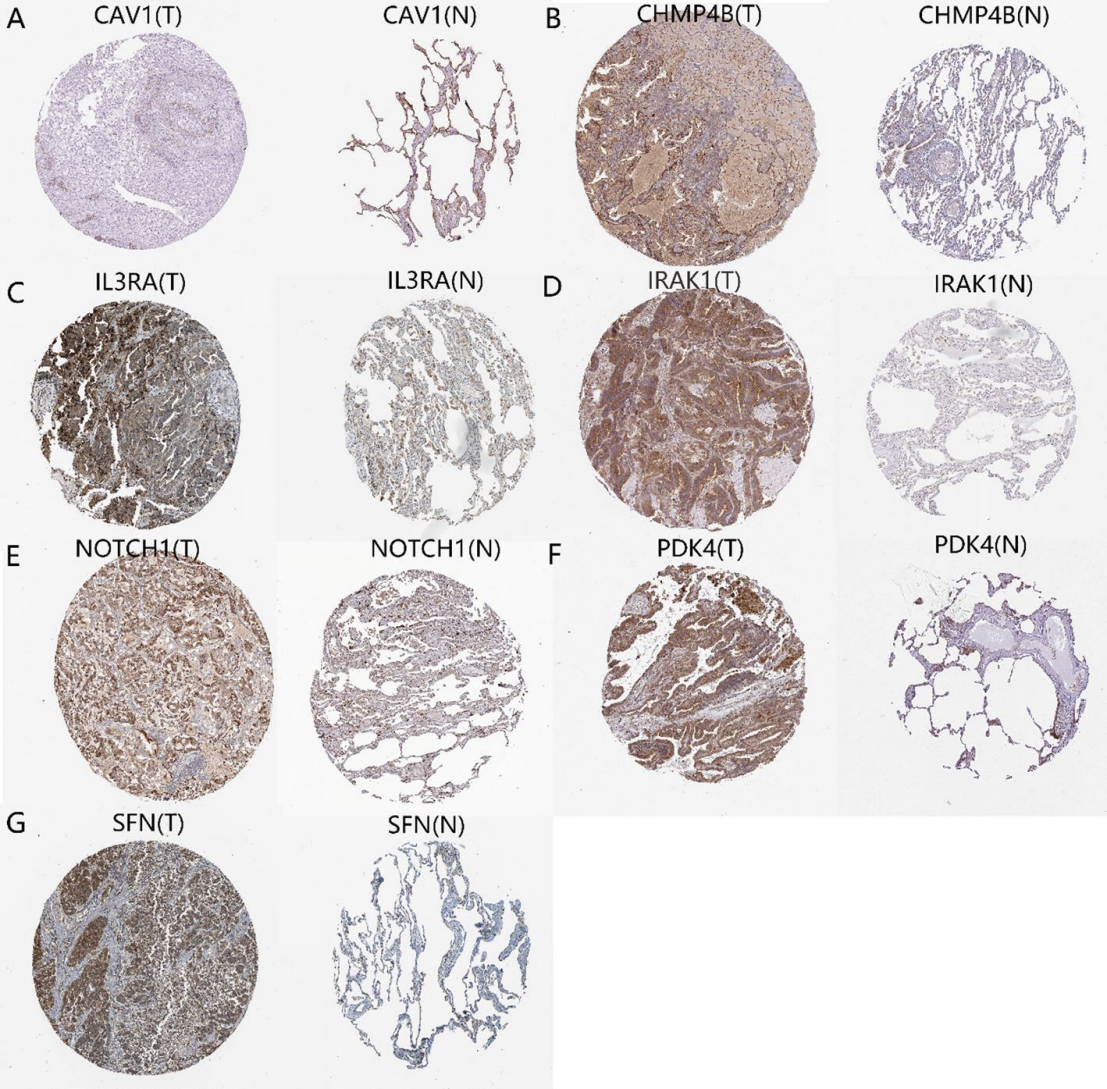
Immunohistochemical analysis of PANoptosis-related DEGs in Lung Cancer. **A** CAV1, **B** CHMP4B, **C** IL3RA, **D** IRAK1, **E** NOTCH1, **F** PDK4, **G** SFN

Immunohistochemical staining images of the relevant genes in cancer and normal tissues were downloaded from the HPA database to observe the differential expression of 7 out of the 8 PANoptosis-related DEGs (excluding DAPK2, as its data were not available in this database) across different tissue samples. As shown in Fig. 2, the expression of these 7 differential genes in normal tissue samples and lung cancer tissue samples exhibited significant differences, consistent with our preliminary analysis results.

### Enrichment analysis of DEGs

**Fig. 3:**
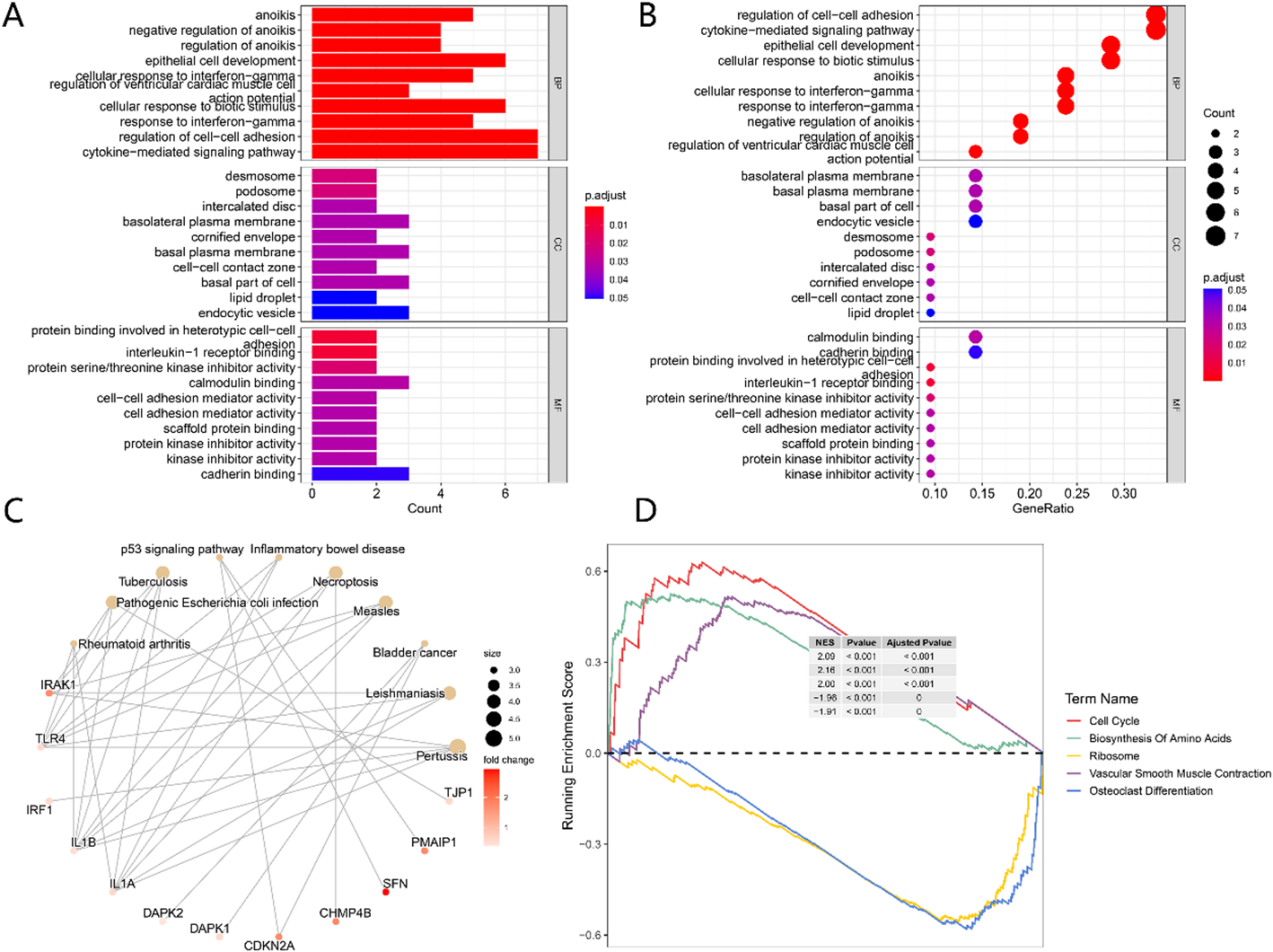
Presentation of GO Enrichment and GSEA Analysis Results of PANoptosis-related DEGs in Lung Cancer. **A**, **B** GO Enrichment Analysis Results, Featuring the Top 10 Terms for Each Branch. **C** Visualization of the Association Between Genes and Functional Annotations in GO Enrichment Analysis. **D** Display of the Top 5 GSEA Analysis Results

GO enrichment analysis was conducted on 8 PANoptosis-related DEGs in lung cancer. As shown in Fig. 3A, B, these 8 genes were enriched in biological processes such as regulation of cell-cell adhesion and cytokine-mediated signaling pathway. The most relevant cellular components were basolateral plasma membrane, etc., and the most relevant molecular functions were calmodulin binding, cadherin binding, etc.

Figure 3C presents the network relationships between genes and pathways among the top ten results of the GO enrichment analysis. KEGG pathway enrichment analysis revealed enrichment in Necroptosis and Pertussis signaling pathways. Figure 3D displays the top 5 GSEA enrichment analysis results, indicating that the differential genes were highly enriched in processes such as Cell Cycle, Biosynthesis Of Amino Acids, and Vascular Smooth Muscle Contraction, while Ribosome and Osteoclast Differentiation were significantly inhibited.

### Immune cell infiltration in single cell data

**Fig. 4:**
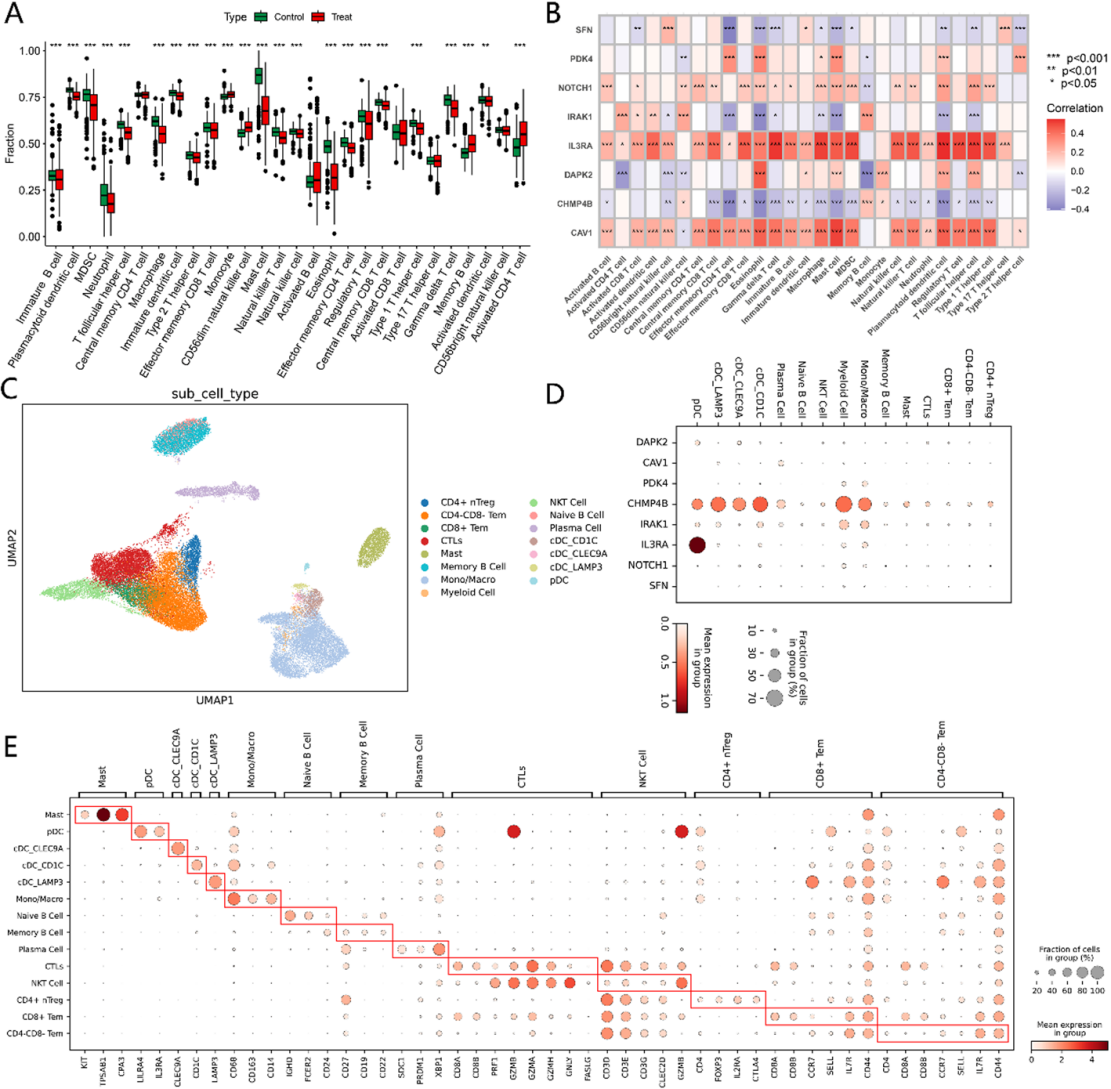
Analysis of Immune Cell Profiles in Lung Cancer Samples and Associated Gene Expression. **A** Boxplot of Immune Cell Scores Across Lung Cancer Samples. **B** Correlation Analysis Between Feature Gene Expression and Immune Cell Scores. **C** UMAP Analysis of Lung Cancer Single-Cell Data. **D** Expression of PANoptosis Target Genes in Immune Cells. **E** Annotation of Cell Types Using Marker Genes

Immune infiltration scores were assigned to all samples using the ssGSEA method, and the results showed significant differences in the infiltration levels of most immune cells between the normal and cancer groups (Fig. 4A). Subsequently, the correlations between immune infiltration scores and feature genes were assessed, revealing a high correlation between the 8 PANoptosis-related DEGs, including DAPK2, CAV1, PDK4, and others, and immune cells (Fig. 4B). Figure 4C displays the UMAP plot of the single-cell analysis results for lung cancer. As shown in Fig. 4D, the expression of PANoptosis-related DEGs in immune cell types was analyzed, revealing high expression of CHMP4B in Myeloid Cell, Mono/Macro, and cDC_CD1C, and high expression of IL3RA in pDC. As shown in Fig. 4E, we used KIT, TPSAB1, CPA3 to mark Mast Cells, LILRA4, IL3RA to mark pDC, and CD68, CD163, CD14 to mark monocytes/macrophages, etc.

**Fig. 5:**
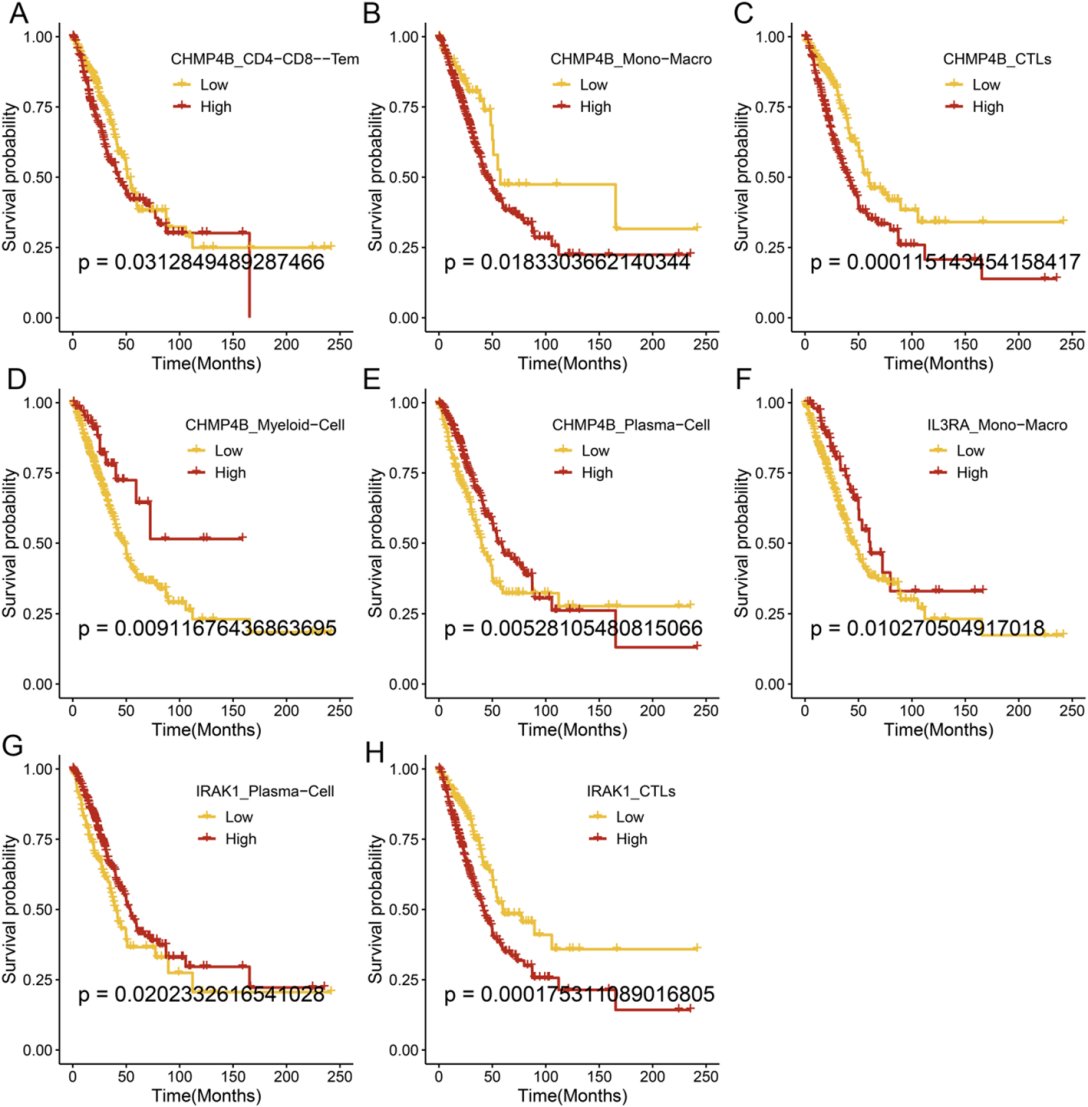
Survival analysis based on the expression of PANoptosis-related DEGs in different immune cells in LUAD

Subsequently, based on the correlations between PANoptosis target genes and immune cells shown in Fig. 4B, the expressions of CHMP4B and IRAK1 in various immune cell types were analyzed in subgroups. As shown in Fig. 5A, patients with high CHMP4B expression in CD4+/CD8 + T cells exhibited lower survival rates than those with low expression in lung adenocarcinoma (*p* = 0.0313). As shown in Fig. 5B-E, patients with high CHMP4B expression in monocytes/macrophages (*p* = 0.0183) and CTLs (*p* = 0.0001) had lower survival rates than those with low expression in lung adenocarcinoma, whereas patients with high CHMP4B expression in Myeloid-Cell (*p* = 0.0091) and Plasma-Cell (*p* = 0.0053) had higher survival rates. Additionally, as shown in Fig. 5F–H, patients with high IL3RA in monocytes/macrophages (*p* = 0.0103) and IRAK1 in Plasma-Cell (*p* = 0.0202) had higher survival rates, whereas patients with high IRAK1 expression in CTLs cells had lower survival rates (*p* = 0.0002).

**Fig. 6:**
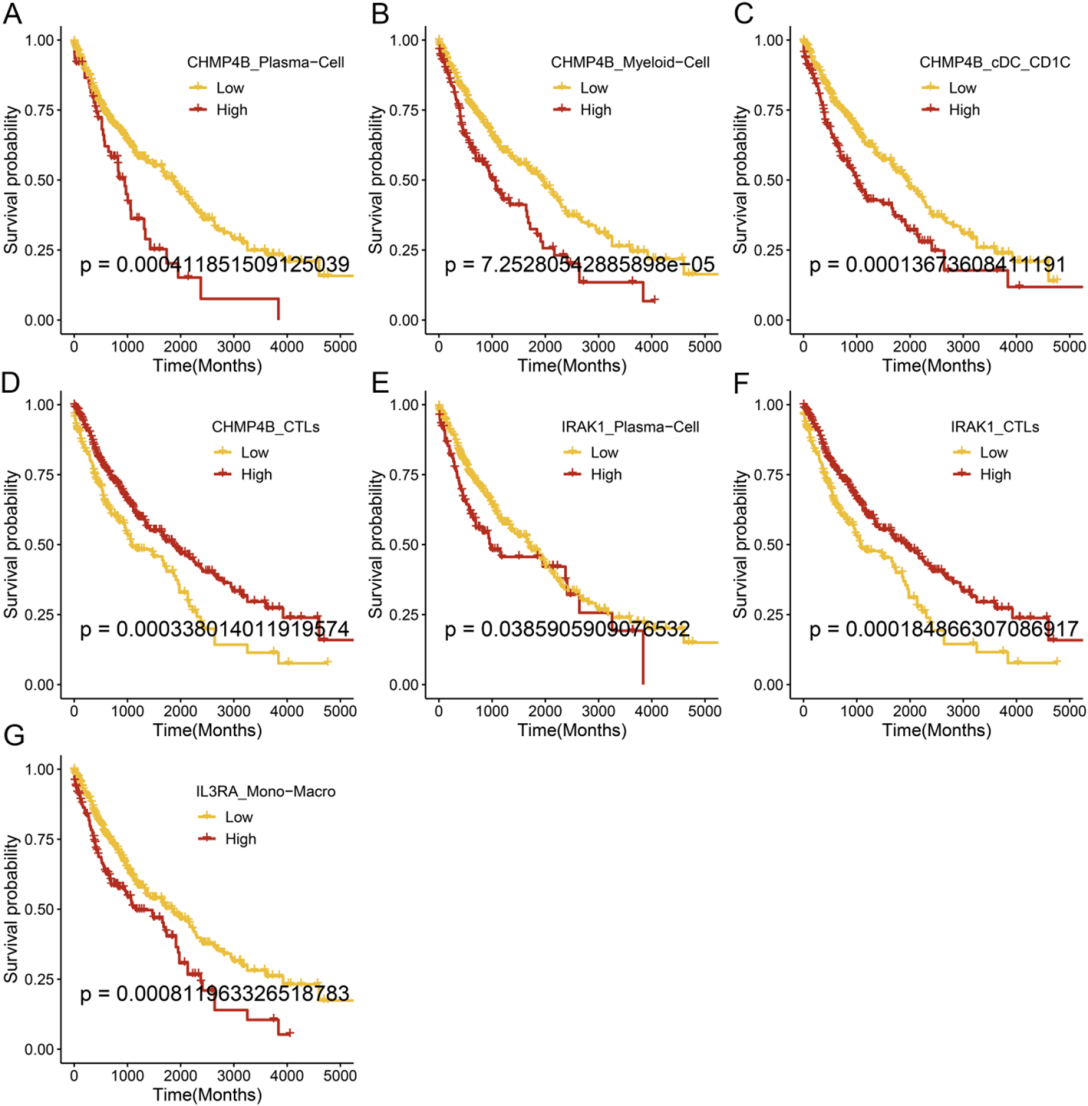
Survival analysis based on the expression of PANoptosis-related DEGs in different immune cells in LUSC

In lung squamous cell carcinoma, patients with high CHMP4B expression in Plasma-Cell, Myeloid-Cell, and cDC_CD1C, high IRAK1 in Plasma-Cell, and high IRAK1 expression in monocytes/macrophages had lower survival rates than those with low expression (Fig. 6A–C, E, G). On the contrary, patients with high CHMP4B and IRAK1 expression in CTLs had higher survival rates than those with low expression (Fig. 6D–F).

### Drug enrichment analysis and molecular docking

**Fig. 7:**
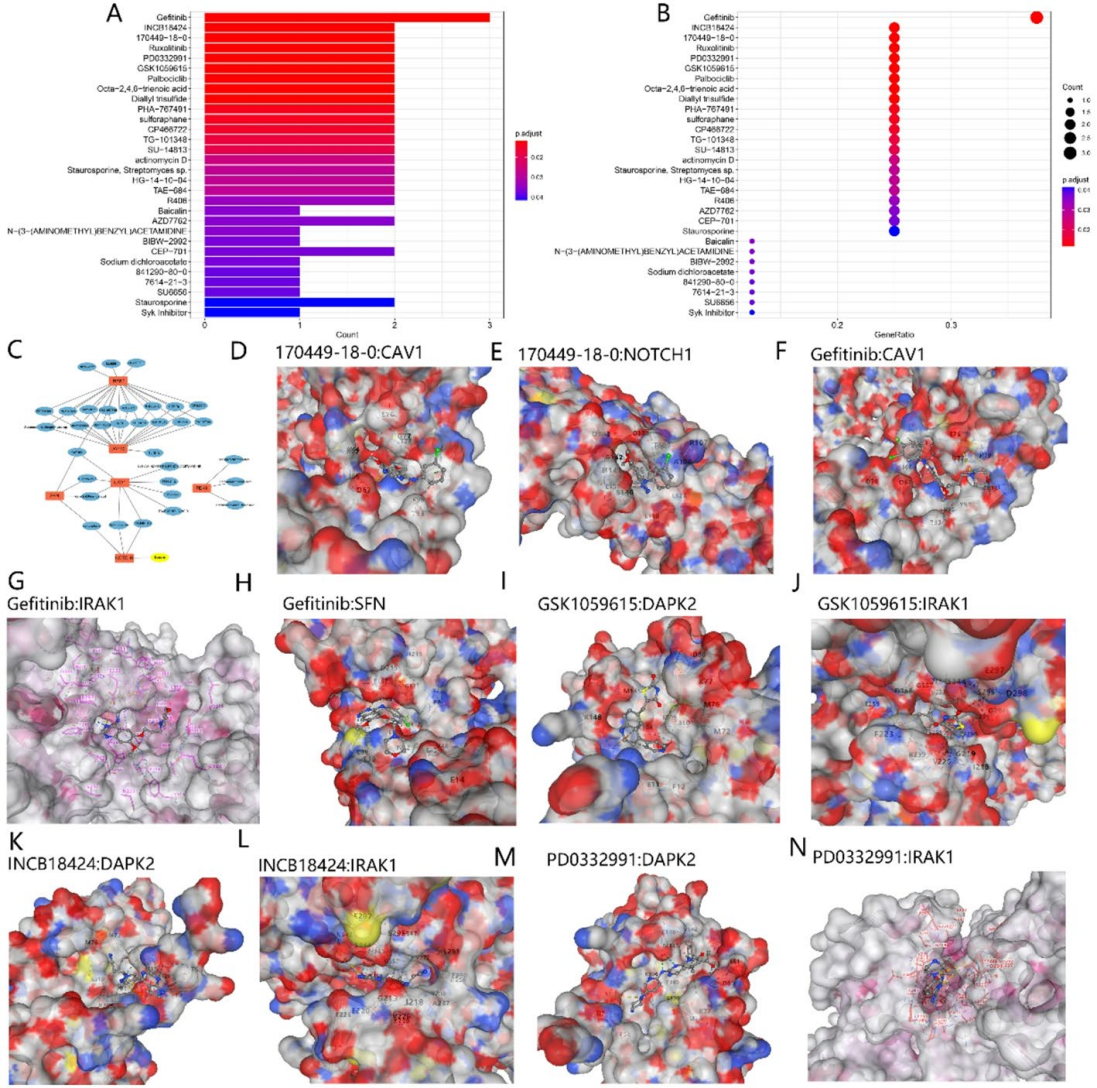
Visualization of Drug Enrichment Analysis and Molecular Docking Results for PANoptosis-related DEGs in Lung Cancer. **A**, **B** Drug Enrichment Analysis: Bar Chart (**A**) and Bubble Chart (**B**). **C** Drug-Gene Interaction Network. **D**–**N** Molecular Docking Results of Selected Drugs with Target Genes (CAV1, Notch1, SFN, DAPK2, and IRAK1)

Drug regulatory enrichment analysis was performed on the 8 PANoptosis feature genes, revealing significant correlations between drugs such as Gefitinib, INCB18424, 170449-18-0, Ruxolitinib, PD0332991, GSK1059615, and these 8 genes (Fig. 7A, B). Among them, Gefitinib was associated with CAV1, IRAK1, and SFN genes (Fig. 7C). Subsequently, the 3D structural proteins of CAV1, NOTCH1, IRAK1, DAPK2, and the structural information of the drugs were downloaded, and molecular docking was performed between Gefitinib, 170449-18-0, and these three genes. Interactions were observed between 170449-18-0 and CAV1, NOTCH1 (Fig. 7D, E). Gefitinib interacted with CAV1, IRAK1, and SFN (Fig. 7F–H). GSK1059615 and INCB18424 interacted with DAPK2 and IRAK1 (Fig. 7I–L). PD0332991 interacted with DAPK2 and IRAK1 (Fig. 7M, N).

### Classification model and machine learning

**Fig. 8:**
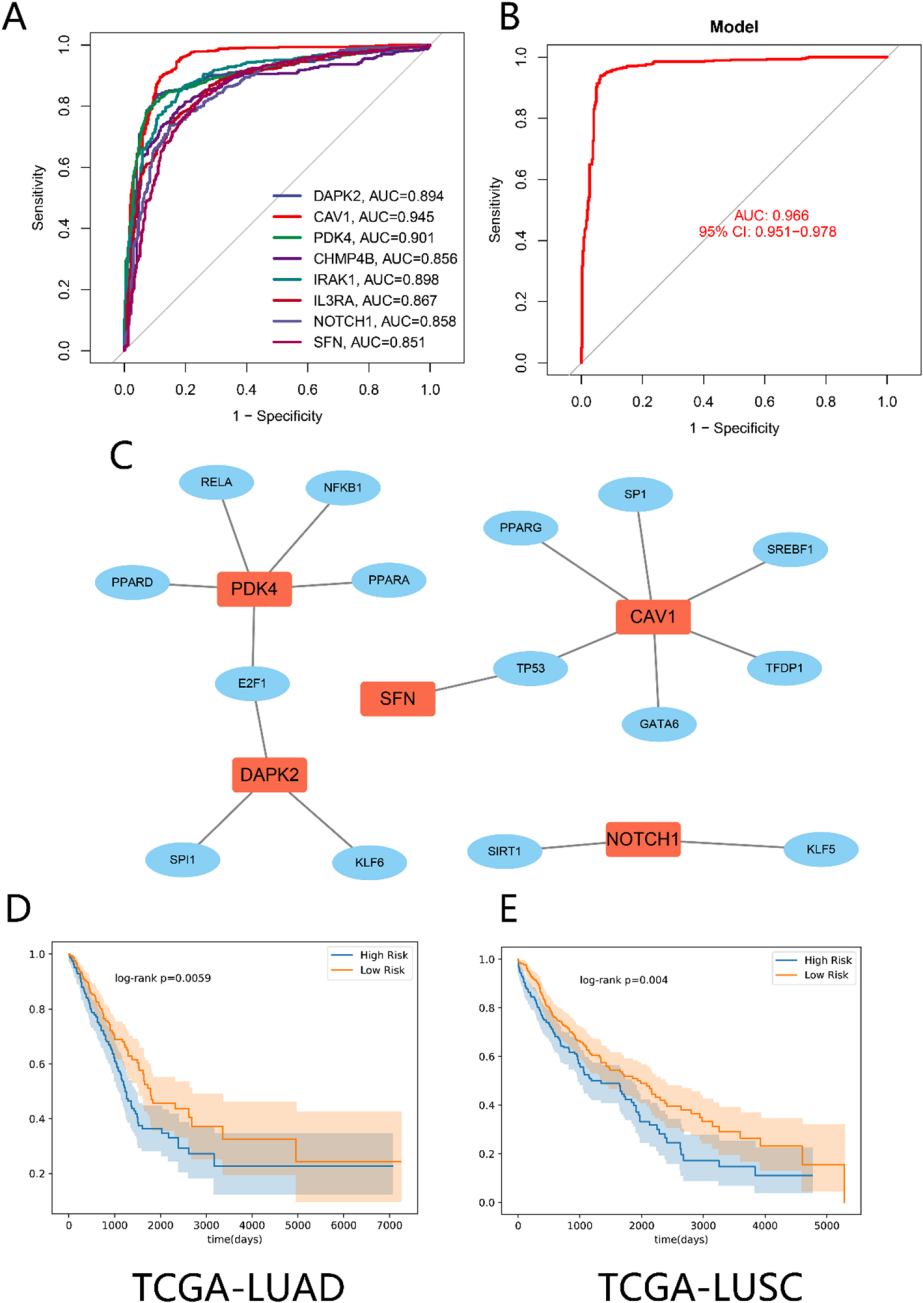
ROC analysis and regulatory network construction of PANoptosis-related DEGs in lung cancer. **A** ROC Curve for Single Gene Analysis. **B** ROC Curve for Logistic Regression Model Based on 8 PANoptosis-related DEGs. **C** RNA-Binding Protein Network Constructed with the 8 Genes. **D** Prognostic Analysis of LUAD. **E** Prognostic Analysis of LUSC

We assessed the diagnostic potential of each of the 8 PANoptosis-related DEGs in predicting lung cancer status based on the expression of each single gene. As shown in the receiver operating characteristic (ROC) curves in Fig. 8A, all eight genes demonstrated high predictive accuracy for distinguishing cancer from non-cancer samples, with area under the curve (AUC) values exceeding 0.85, indicating their strong diagnostic value. Simultaneously, a logistic regression model incorporating these eight genes was constructed to predict cancer status, achieving an AUC value of 0.966 (Fig. 8B). As shown in Fig. 8C, for the 8 feature genes, we downloaded their RNA-binding proteins (RBPs) from the ENCORI database and constructed an RBP network. We also constructed a transcriptional regulatory network using transcription factor data related to these 8 feature genes from the TRRUST database. Finally, based on the above results, patients were divided into high-risk and low-risk groups. As shown in Fig. 8D, the survival rate of patients in the high-risk group was significantly lower than that in the low-risk group for lung adenocarcinoma (*p* = 0.0059). Similarly, as shown in Fig. 8E, the survival rate of patients in the high-risk group was significantly lower than that in the low-risk group for lung squamous cell carcinoma (*p* = 0.0040). Nomogram-related analyses are presented in Supplementary Fig. 3.

### Expression validation of PANoptosis-related DEGs by qRT-PCR

**Fig. 9:**
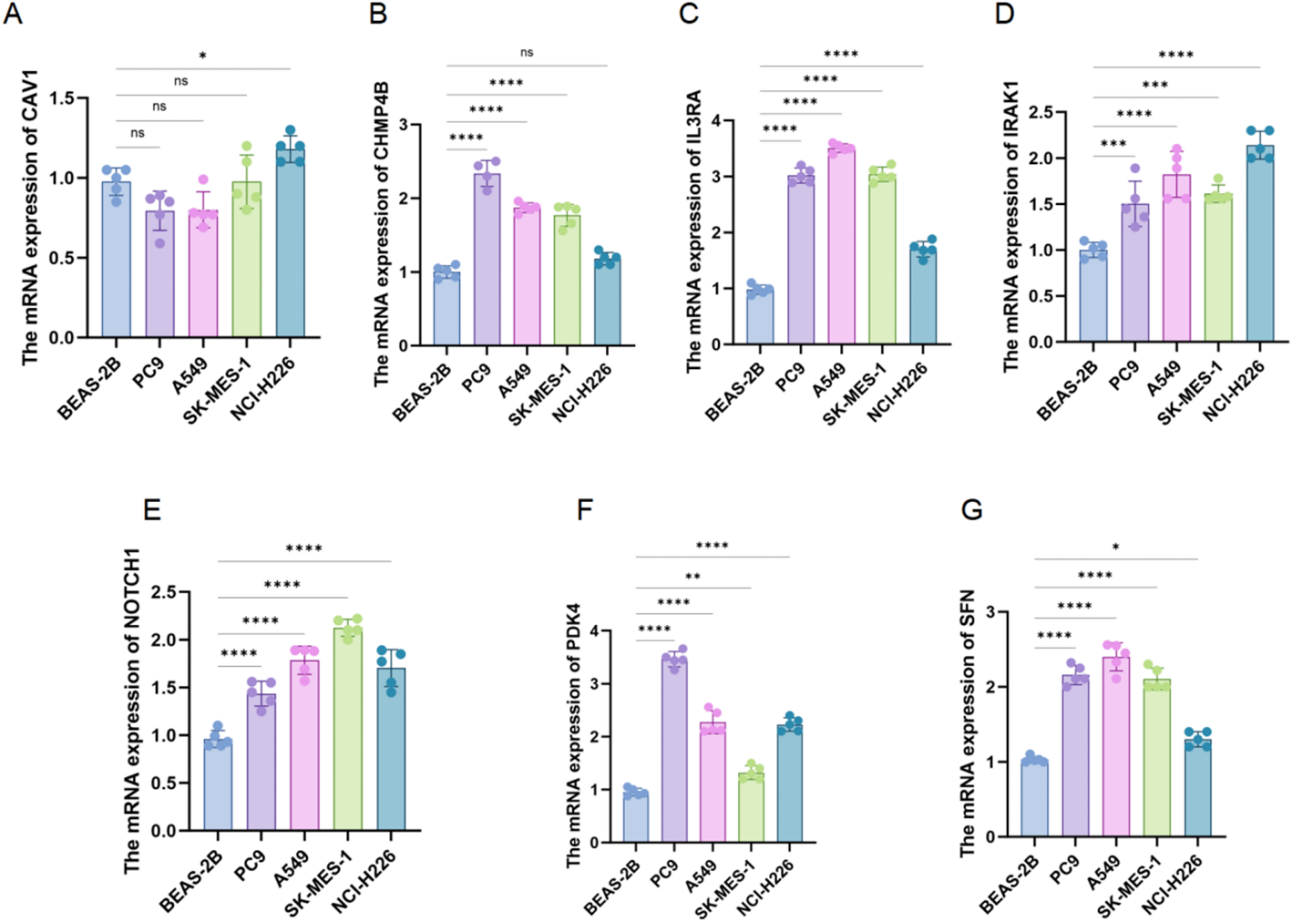
The mRNA Expression Levels of Candidate PANoptosis-related Genes in normal and NSCLC cell lines. Quantitative RT-PCR analysis was performed to measure the mRNA expression of **A** CAV1, **B** CHMP4B, **C** IL3RA, **D** IRAK1, **E** NOTCH1, **F** PDK4, and **G** SFN in BEAS-2B (normal bronchial epithelial cells), PC9, A549 (lung adenocarcinoma cells), SK-MES-1, and NCI-H226 (lung squamous cell carcinoma cells). Data are shown as mean ± SD from at least three biological replicates. Statistical significance was assessed using one-way ANOVA with multiple comparisons. *p < 0.05, **p < 0.01, ***p < 0.001, ****p < 0.0001, ns = not significant

To experimentally validate the differential expression of these candidate PANoptosis-related DEGs between tumor and non-tumor cells, we performed qRT-PCR for examining the mRNA levels of CAV1, CHMP4B, IL3RA, IRAK1, NOTCH1, PDK4, and SFN across one normal bronchial epithelial cell line (BEAS-2B) and four non-small cell lung cancer (NSCLC) cell lines (PC9 and A549: LUAD; SK-MES-1 and NCI-H226: LUSC). Compared to BEAS-2B, the mRNA expression of most candidate genes was significantly upregulated. Specifically, CHMP4B was markedly elevated in PC9, A549, and SK-MES-1 (*p* < 0.0001), while IL3RA was robustly upregulated across all cancer cell lines (*p* < 0.0001). Both IRAK1 and NOTCH1 were consistently overexpressed in all cancer cells (*p* < 0.001 or *p* < 0.0001). PDK4 expression peaked in PC9 cells and was lower in the other lines, and SFN expression was significantly higher in PC9, A549, and SK-MES-1. In contrast, CAV1 was significantly upregulated only in the squamous carcinoma cell line NCI-H226 (*p* < 0.05). Notably, a distinct expression pattern emerged across histological subtypes: most genes were upregulated in adenocarcinoma-derived lines (PC9 and A549), whereas NCI-H226 exhibited a different profile with lower levels of IL3RA, CHMP4B, and SFN but elevated CAV1 (Fig. 9). These results confirm the differential expression of PANoptosis-related genes in lung cancer cells and suggest the existence of subtype-specific transcriptional signatures that may reflect distinct roles in tumor progression.

## Discussion

The present study identified eight PANoptosis-related genes (DAPK2, CAV1, PDK4, IL3RA, NOTCH1, CHMP4B, IRAK1, SFN) associated with lung cancer through the integration of lung cancer transcriptome data from the GEO and TCGA databases, coupled with single-cell sequencing analysis. These genes exhibited significant differences in expression levels between lung cancer tissues and normal tissues and were closely related to patient prognosis. Enrichment analysis revealed that these genes were primarily involved in biological processes such as cell adhesion, cytokine signaling pathways, necrosis, and the cell cycle, suggesting that they may influence the initiation and progression of lung cancer by regulating these crucial pathways. For instance, DAPK2, which was significantly downregulated in lung cancer tissues, plays a pivotal role in cell adhesion and cell death. It may inhibit lung cancer invasion and metastasis by suppressing tumor cell adhesion and migration [16]. CAV1, which was also downregulated in lung cancer tissues, is crucial for cell cycle regulation and cell survival. The absence of CAV1 may promote the proliferation and survival of lung cancer cells [17, 18]. CHMP4B, which was markedly upregulated in lung cancer tissues, plays a significant role in the cellular endomembrane system and cell death [19, 20]. The high expression of CHMP4B may enhance the survival and immune evasion of lung cancer cells [21]. The discovery of these genes provides novel insights into the molecular mechanisms underlying lung cancer. The abnormal expression of PANoptosis-related genes may impact the initiation and progression of lung cancer through various mechanisms, including the regulation of key biological processes such as the cell cycle, cell adhesion, and cell death [22, 23]. Additionally, the expression levels of these genes are closely related to patient prognosis, suggesting their potential as biomarkers for the diagnosis and prognosis assessment of lung cancer.

Immune infiltration plays a crucial role in the initiation and progression of lung cancer [12, 24, 25]. In this study, the correlation between PANoptosis genes and immune cell infiltration was analyzed using the ssGSEA method. It was found that genes such as DAPK2, CAV1, and PDK4 were highly correlated with the infiltration levels of various immune cells. For example, CHMP4B was highly expressed in myeloid cells, monocytes/macrophages, and dendritic cells, and its expression was positively correlated with the infiltration levels of these immune cells. This indicates that CHMP4B may influence the immune microenvironment of lung cancer by regulating immune cell infiltration [26]. Further single-cell analysis revealed that high expression of CHMP4B in myeloid cells and dendritic cells was associated with poor prognosis, while high expression in plasma cells was associated with favorable prognosis. This suggests that the role of CHMP4B may be heterogeneous across different immune cells, and its specific mechanism of action in lung cancer warrants further investigation.

Moreover, high expression of IRAK1 in plasma cells was associated with favorable prognosis, whereas high expression in cTLS cells was associated with poor prognosis. This also indicates that the role of IRAK1 may differ across different immune cells [27–29]. These findings suggest that PANoptosis genes may affect the immune microenvironment of lung cancer and patient prognosis by regulating immune cell infiltration and function. The infiltration levels and functional states of immune cells are crucial factors determining the response to cancer immunotherapy. Therefore, the role of PANoptosis genes in the lung cancer immune microenvironment warrants further study.

Drug regulatory analysis revealed that several candidate drugs were significantly correlated with the expression of PANoptosis-related DEGs. Notably, Gefitinib—an epidermal growth factor receptor (EGFR) tyrosine kinase inhibitor widely used in lung cancer therapy—showed strong correlations and predicted molecular interactions with key PANoptosis genes including CAV1 and SFN [30–32]. In addition to its established interactions with the aforementioned PANoptosis-related DEGs in suppressing EGFR-driven cell proliferation—for instance, CAV-1 modulates EGFR signaling in breast cancer cells and enhances gefitinib-induced tumor suppression, and may also be linked to autophagy-endocytosis crosstalk—our analysis suggests that gefitinib may also concurrently regulate apoptotic pathways through these PANoptosis-related molecular interactions, thereby exerting anti-tumor effects [33–36]. Studies indicate that CAV-1 plays a role in regulating drug sensitivity to gefitinib in lung cancer; knockdown of CAV-1 significantly enhances lung cancer sensitivity to gefitinib by downregulating EGFR phosphorylation. Moreover, CAV1 acts as a regulator of multiple cell death modalities and immune signaling, while SFN is associated with apoptosis and tumor cell proliferation regulation. Both, along with gefitinib, are closely related to key apoptotic pathways such as mitochondrial function regulation and MAPK signaling. CAV1 or SFN may interact with the mitochondrial apoptotic pathway of gefitinib, and presumably, inhibiting CAV1 or SFN could enhance the pro-apoptotic effect of gefitinib by restoring mitochondrial function or blocking the AKT/EGFR signaling. Therefore, by targeting these genes, gefitinib may influence the cellular apoptotic signaling network and alter the balance between cell survival and inflammatory cell death in tumor cells. Similarly, the compound 170449-18-0 displayed interaction targets with CAV1 and NOTCH1, another key player in cell fate decisions and inflammatory responses, suggesting its potential to exert an anti-lung cancer effect by regulating the expression of these genes to perturb PANoptosis-associated pathways. These findings highlight promising pharmacological avenues for targeting the PANoptosis axis in lung cancer. However, it is important to note that this drug analysis was based on computational predictions from public databases and lacks experimental validation. Future studies are necessary to functionally validate these drug-gene interactions and to explore their therapeutic potential in modulating PANoptosis for lung cancer treatment.

In this study, random forest and Lasso regression models were constructed to assess the value of PANoptosis genes in predicting lung cancer occurrence and prognosis. The results indicated that these eight PANoptosis genes could effectively predict whether a sample was cancerous, with AUC values above 0.85. Furthermore, when the eight genes were fitted into a logistic regression model to predict cancer status, the AUC reached 0.966. This suggests that these PANoptosis genes have good predictive value and could serve as potential lung cancer biomarkers. Further survival analysis revealed that when patients were divided into high-risk and low-risk groups, the survival rate of the high-risk group was significantly lower than that of the low-risk group, indicating that these PANoptosis genes could be used as prognostic indicators for lung cancer. Additionally, by constructing RBP and transcriptional regulatory networks, we elucidated the regulatory mechanisms of PANoptosis genes in lung cancer. These findings provide a theoretical basis for the development of PANoptosis gene-based tools for lung cancer diagnosis and prognosis. However, this study had a limited sample size and lacked an independent validation cohort. Therefore, future studies are needed to validate the predictive value of these PANoptosis genes in larger sample sizes and explore their feasibility in clinical applications.

Although this study systematically identified the key genes of PANoptosis related to lung adenocarcinoma and explored their clinical significance, there are still some limitations, such as the lack of experimental verification from patient tissue samples and direct functional experimental evidence. Future research can utilize these experimental methods to validate the correlation between PANoptosis gene expression and the level of specific immune cell infiltration in independent patient cohorts. Future research efforts should focus on using experimental biological methods to verify the biological functions of these PANoptosis genes and evaluate their application value as diagnostic, prognostic markers, and therapeutic targets in a wider range of clinical samples, thereby providing new theoretical and practical basis for precision medicine of lung adenocarcinoma.

## Conclusion

Through the integration of multi-omics data, this study comprehensively analyzed PANoptosis-related genes in lung cancer, revealing their mechanisms of action in the initiation and progression of lung cancer and exploring their feasibility as potential biomarkers and therapeutic targets. Our findings indicate that PANoptosis genes play crucial biological roles in lung cancer and could serve as potential biomarkers and therapeutic targets. Future studies are needed to experimentally validate the functions of these PANoptosis genes and explore their potential in clinical applications.

## Supplementary Information

Supplementary Material 1. Fig.1 Demonstration of Batch Correction Effects on Lung Cancer DataA. Principal Component Analysis of Data Before Batch Correction. B. Principal Component Analysis of Data After Batch Correction

Supplementary Material 2. Fig.2 Supplementary Analysis of PANoptosis-Related Feature Gene Selection in Lung Cancer and Chromosomal Location of These Genes

Supplementary Material 3. Fig3. Data related to nomogram

Supplementary Material 4.

## Data Availability

The data that support the findings of this study are available from the corresponding author upon reasonable request.
