# Supplementary figures and images for: Identification of PANoptosis-related genes in lung cancer and investigation of their role mechanisms in the immune microenvironment
Source: Discov Oncol. 2026 Feb 28;17:536. doi: 10.1007/s12672-026-04739-1 (PMC13057117; doi:10.1007/s12672-026-04739-1)

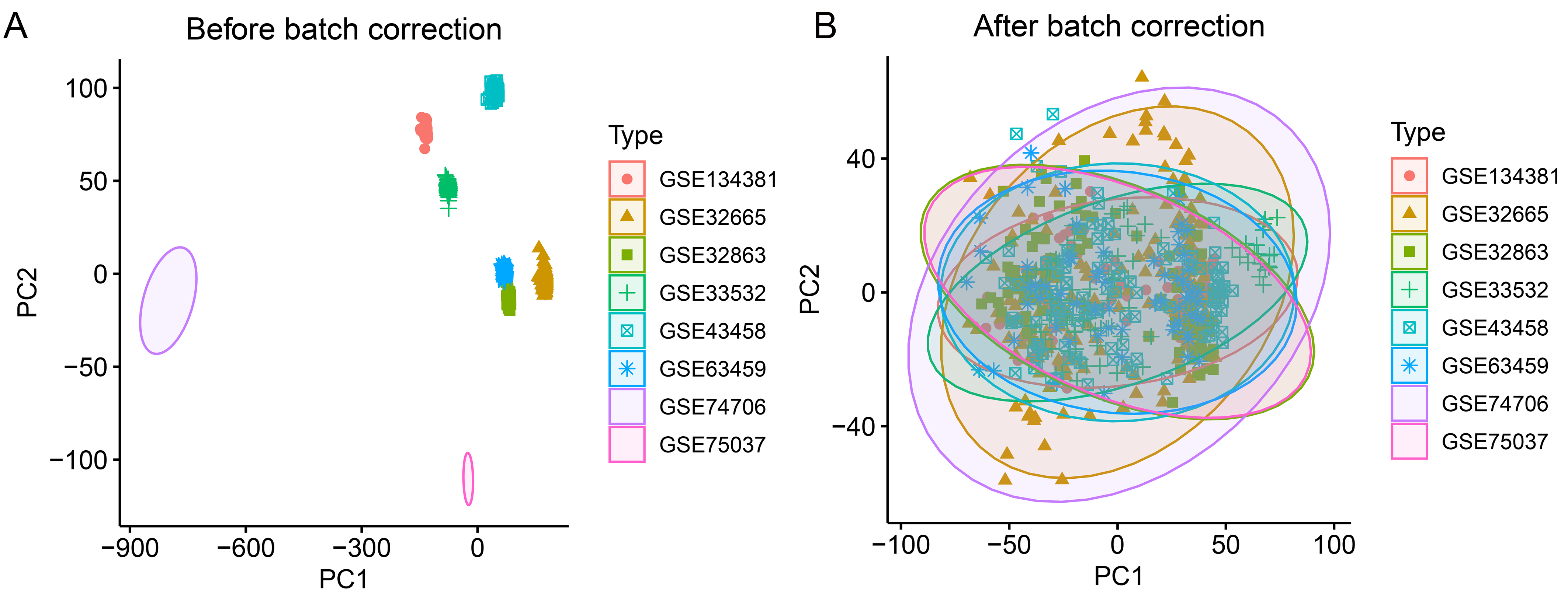

Supplement: Supplementary file 2 — Supplementary Material 2. Fig.2 Supplementary Analysis of PANoptosis-Related Feature Gene Selection in Lung Cancer and Chromosomal Location of These Genes [file 12672_2026_4739_MOESM2_ESM.tif]

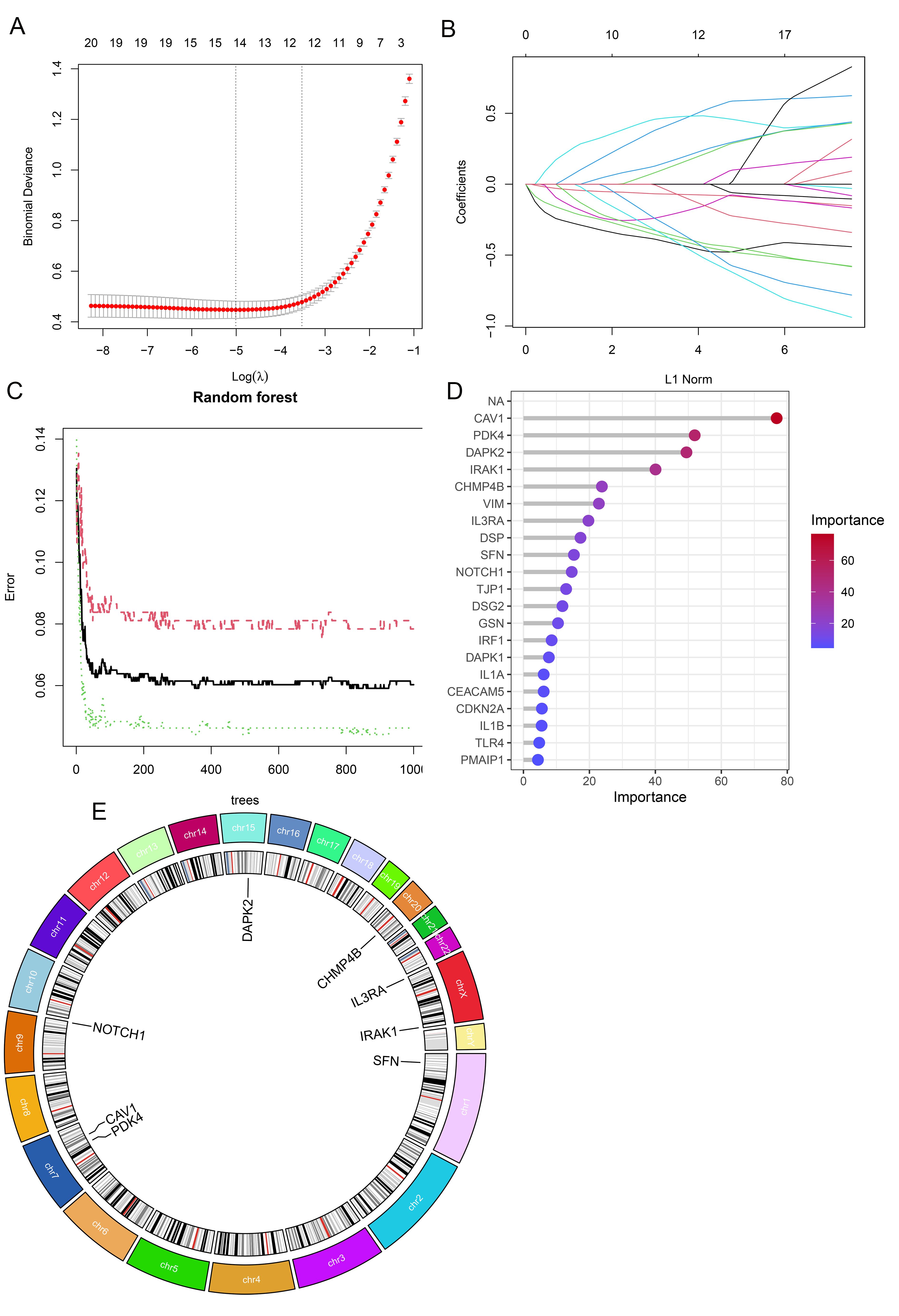

Supplement: Supplementary file 3 — Supplementary Material 3. Fig3. Data related to nomogram [file 12672_2026_4739_MOESM3_ESM.tif]

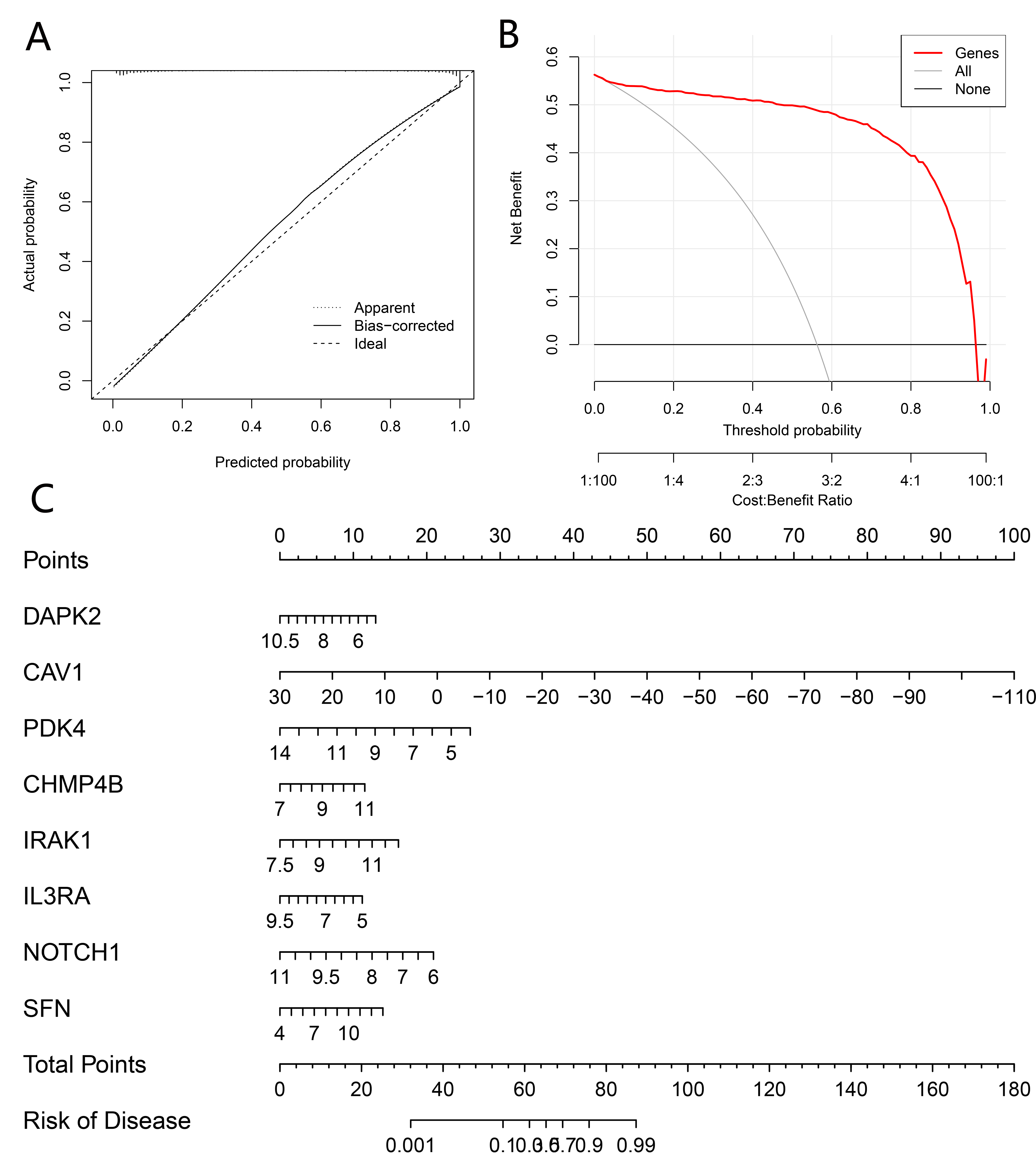

Supplement: Supplementary file 4 — Supplementary Material 4. [file 12672_2026_4739_MOESM4_ESM.tif]
